# Photophysiologically active green, red, and brown macroalgae living in the Arctic Polar Night

**DOI:** 10.1038/s41598-023-44026-5

**Published:** 2023-10-20

**Authors:** Natalie Summers, Glaucia M. Fragoso, Geir Johnsen

**Affiliations:** 1 Centre for Autonomous Marine Operations and Systems (AMOS), Trondheim Biological Station, Department of Biology, Norwegian University of Science and Technology (NTNU), Trondheim, Norway; 2 University Centre in Svalbard (UNIS), Longyearbyen, Norway

**Keywords:** Marine biology, Biological techniques

## Abstract

Arctic macroalgae species have developed different growth strategies to survive extreme seasonal changes in irradiance in polar regions. We compared photophysiological parameters such as the light saturation parameter (E_k_) and pigment composition of green, red, and brown macroalgae collected in January (Polar Night) and October 2020 (end of the light season). Macroalgae in January appeared healthier (morphologically) and had longer lamina (new growth) than those in October. E_K_ values for red, and brown algae were higher with lower maximum quantum yield of PS II fluorescence (F_v_/F_m_) in January versus October. Furthermore, in January, new tissues in kelp species had higher E_K_ than the older tissue. Higher E_K_ and lower F_v_/F_m_ during the Polar Night indicates that the photosynthetic apparatus is active but slow. Furthermore, we discuss Chlorophyll (Chl) a emission spectra under blue and green excitation light to determine the ratio of Chl a in photosystem II (PS II) vs photosystem I (PS I). Absorbance spectra of *P. palmata* was used to interpret the emission spectra. The observed spectral shifts in the absorbance and reflectance spectra of different macroalgae is discussed. Photophysiological methods provide health information complementary to future mapping and monitoring of macroalgae. These results reveal that macroalgae grow new tissue in darkness.

## Introduction

Macroalgae are important primary producers of coastal ecosystems, providing substrate, food, and shelter for organisms of different trophic levels^1,2^. Primary production in macroalgae is primarily controlled by irradiance (E) in the spectral range of 400 to 700 nm (E_PAR_), but other key environmental variables, such as temperature and nutrient availability also modulate the rate of photosynthesis^3–6^. In Svalbard (longitude of 79°N), the winter solstice on December 22nd marks the darkest day of the Polar Night, with December and January being the two darkest months with monthly average E_PAR_ of 67 E^−06^ μmol photons m^−2^ s^−1^ in December 2019 and 234 E^−06^ μmol photons m^−2^ s^−1^ in January 2020. On the other hand, the monthly average E_PAR_ during the summer solstice in June 2020 was 392 μmol photons m^−2^ s^−1^ when measured from the “ArcLight” observatory located about 4 km west of Ny-Ålesund (78.9◦N, 11.9◦E)^7^. Despite the extreme E values, some Arctic macroalgae have been reported to grow in low light conditions and tolerate darkness for longer periods (up to 18 months)^8^. For instance, a study conducted in October 2016 and February 2017 on the eco-physiology of *Laminaria solidungula*, an endemic Arctic brown macroalgae species, and *Saccharina latissima*, a boreal species, suggested that kelp species can maintain their photosynthetic capabilities during the Polar Night^2,9^. *Laminaria solidungula* can store energy gained from photosynthesis to grow during the winter darkness^9–12^. *Saccharina latissima* can delay its growth period until the late spring/summer when light is available^2,13,14^. Growth and survival of these macroalgal species during the Polar Night rely, known so far, on the usage of stored sugar compounds accumulated through photosynthesis during previous summers^8,9^. While it has been shown that many polar organisms (e.g. zooplankton and fish) are highly sensitive to small changes in light intensity during the Polar Night^15,16^, little is known about the photobiological responses of macroalgae during this period of extreme darkness.

Photophysiological responses of macroalgae are not only related to light quantity (intensity of E) but also quality (spectral irradiance E (λ)). For example, the kelp forest canopy limits light penetration with a decrease in light intensity and spectral composition (mostly green light available) for species living below, partly resulting in the zonation of macroalgae^8,17,18^. Understanding the underwater light climate (E_PAR_, E (λ)), and photoperiod) is thus crucial for studies on photosynthesis. Irradiance and the corresponding color (E (λ)) available are important factors for light utilization in photosynthesis^19^. During photosynthesis, excitation energy is either absorbed and utilized by light harvesting pigments, dissipated as fluorescence by chlorophyll a (Chl a) or dissipated as heat^20^. Different pigment composition in macroalgae determines their classification into green, brown, and red algae as well as which wavelengths they can absorb and utilize^21^. The pigment concentrations also affect the ability of macroalgae to harvest light of different wavelengths and is thus linked to their photosynthetic ability. Each macroalgal light harvesting pigment has different *in-vivo* light absorption characteristics with chlorophylls absorbing in blue (400-500 nm) and red (600-700 nm), carotenoids absorbing mostly in 400–530nm range and phycobiliproteins absorbing in the blue-green to green range at 500-570 nm^2^. Studying spectral light utilization of algae can explain how they pre-condition their photosynthetic apparatus before light increasingly and rapidly becomes available in late winter/spring^22^.

Increasing temperatures, sedimentation rates and decreasing sea ice cover over the past decade in Kongsfjorden, Svalbard, have altered the underwater light climate, such as darkening of the fjord in summer^23,24^ and resulted in changes in the vertical distributions of algae species^2,18^. This highlights the need for more studies on health state, and seasonality of macroalgae. Enabling technologies have led to more efficient ways of *in-situ* monitoring and mapping of kelp forests. In 2004 hyperspectral imaging (HI) from an airplane was used to map and identify the kelp forest in Ny-Ålesund during the light season^25^. More recently, in January 2020, mapping of the kelp forest in Ny-Ålesund was conducted during the Polar Night using underwater hyperspectral imager (UHI) carried by a novel miniature remotely operated vehicle (ROV) system^26^. This study is the first to carry out photobiological and health state assessment of the most common species of green, brown, and red macroalgae during the Polar Night simultaneously with *in-situ* identification and mapping of the biodiversity and areal cover of the same kelp forest using a UHI from an ROV^26^.

In this study, we measured and compared photosynthetic parameters (photosynthetic efficiency α, maximum relative electron transport rate, rETR_max_, the onset light saturation parameter, E_K_, and maximum quantum yield of PS II fluorescence, F_v_/F_m_) of the most common species of green, red, and brown macroalgae from Kongsfjorden during the Polar Night (January) and at the end of the polar light season (October 2020). These measurements were paired with pigment composition of each of the experimental species as well as the in vivo PS II-Chl a emission spectra under green (525 nm) and blue (452 nm) excitation light, using a spectrometer attached to the Diving PAM II. The primary aim of this study was to determine the photophysiological state of old versus new tissues of different macroalgae species to elucidate seasonal differences. Additionally, we provided a first attempt to interpret emission spectra under blue and green excitation light to infer pigment activity and distribution in the photosystems. Finally, we discuss how the photophysiology of macroalgae affect the absorbance and reflectance spectra and how this can be integrated into future habitat mapping using *in-situ* spectral reflectance per image pixel from mini-ROV with UHI.

## Methods and materials

### Study site and conditions

**Figure 1:**
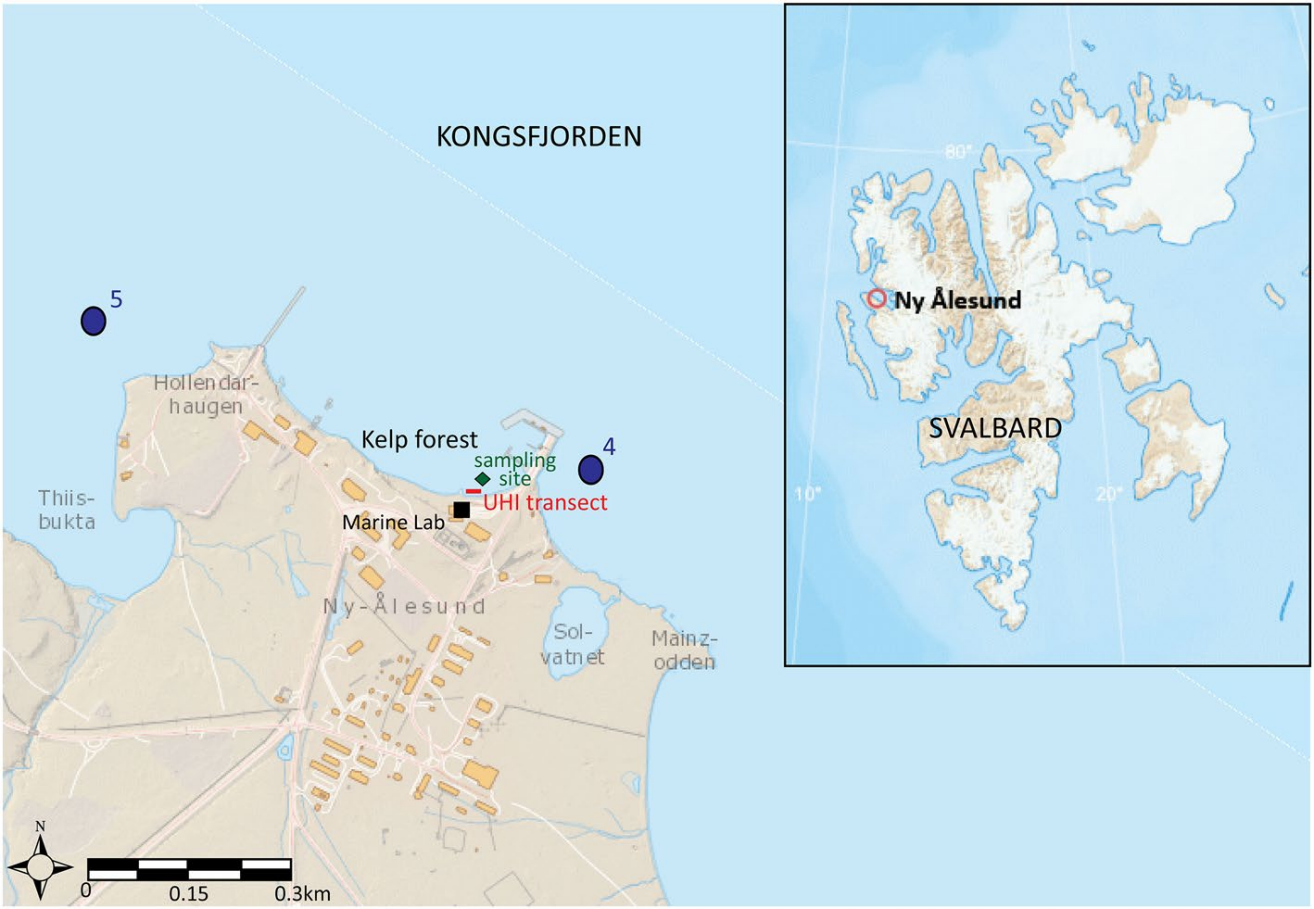
Map showing our study area in Kongsfjorden, Svalbard (78°55′40.0"N 11°55′52.9"E). Localization of our macroalgae sampling site (green diamond) next to the UHI transect (red line) from Summers et al.^26^ and located in front of the marine lab (black square) in Ny-Ålesund. Location of aerial hyperspectral imaging study sites from Volent et al.^25^ are shown as blue dots marked 4 and 5. Base map from https://toposvalbard.npolar.no/, courtesy of the Norwegian Polar Institute.

Macroalgal specimens were sampled between 1 and 3 m depth, 50 m off the coast of the Marine Laboratory in Ny-Ålesund located in Kongsfjorden, Svalbard (78°55′40.0"N 11°55′52.9"E, Fig. 1). Other specimens were also collected on the 11th of January 2020, as part of the Polar Night cruise aboard research vessel Helmer Hanssen, and on the 26th of October 2020 during a research stay in Ny-Ålesund. The bay in front of the Marine Laboratory was between 0.5 to 4 m deep and characterized with rocky and sandy substrate covered with macroalgae. In the shallow areas (closest to shore) ice formed on the beach (ice foot) and corresponding free moving sea ice had caused ice scouring of macroalgae in the upper 1.5 m due to tidal range of approximately 1.3 m (no visible macroalgae). Above water surface irradiances were provided by the ArcLight observatory located about 4 km west of Ny-Ålesund (78.9◦N, 11.9◦E) and using an all-sky camera as irradiance sensor. Specifications of the different light measuring instruments and calibration procedure, as well as data from 2017–2020 were published by Johnsen et al.^7^. Samples from the 11th of January 2020 was part of the Polar Night season with E_PAR_ far below the theoretical threshold to induce actinic activity. The average E_PAR_ above the water surface for January 2020 was 234 E^−06^ μmol photons m^−2^ s^−1^ versus a theoretical irradiance to induce photosynthesis set at 0.01 μmol photons m^−2^ s^−1 ^^7^. The weather was calm and water temperature was at − 1.8°C resulting in the formation of pancake ice on the surface of the bay. The average downwelling E_PAR_ above the water surface in October 2020 was 6.6 μmol photons m^−2^ s^−1^. However, October is also a light transition period when light availability decreases throughout the month with only 44% of hours per month being above E_PAR_ of 0.01 μmol photons m^−2^ s^−1^, inducing photosynthetic activity. On the 26th of October 2020 (day of sample collection), the weather was calm with clear seawater at a temperature of 1.5 to 2°C.

In January 2020, we collected 3 specimens of each of the 5 species of macroalgae by snorkeling; the green alga (Chlorophyceae) *Ulva* sp*.,* the red alga (Rhodophyceae) *Palmaria palmata*, and the brown algae (Phaeophyceae), consisting of the kelp species *Laminaria digitata* (morphologically similar to *Saccharina nigripes*, synonym to *Hedophyllum nigripes*, see the discussion^18,27^), *Alaria esculenta, and Saccharina latissima*. The algal specimens were collected during two sampling sessions, 12 h apart, during which the experimental specimens were kept in collection nets at the collection site and 1m depth. Once back onboard the research vessel Helmer Hanssen, the living specimens were stored in a dark cooling room (4°C) in buckets full of freezing seawater, providing an *in-situ* temperature of − 1.8°C, until experiments were carried out at the same temperature^26^. Specimens were kept in the dark at all times until experiments were conducted. Specimen collection in January 2020 was conducted at the same time as an ROV-based survey using underwater hyperspectral imaging (UHI) [results published in 26]. The UHI-based mapping provided information on biodiversity and abundance of the different macroalgae groups as described by Summers et al.^26^, with the present study providing additional information on the photophysiological state of the species.

**Figure 2:**
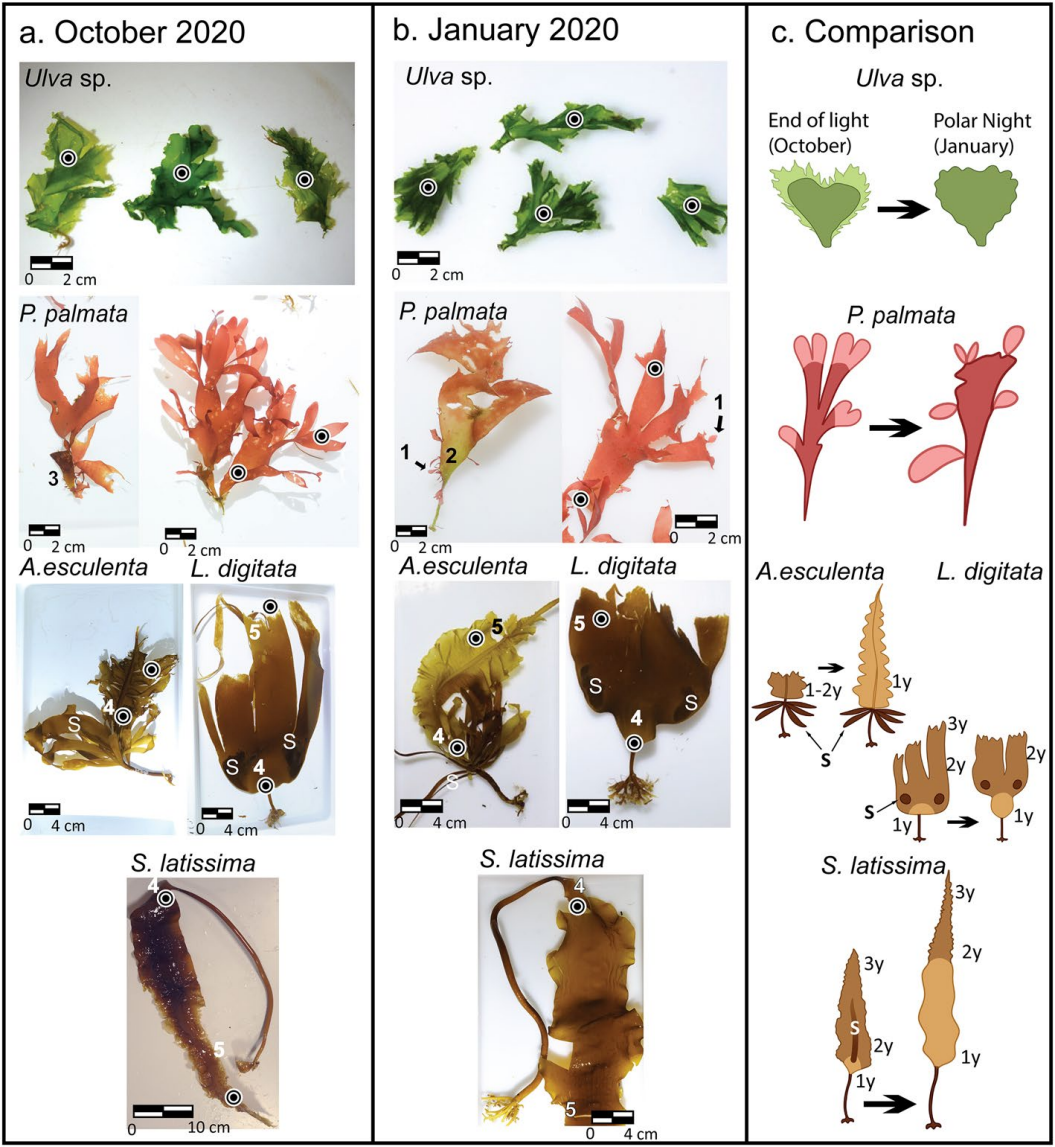
Macroalgal species collected in October (**a**) and January (**b**) 2020 from Kongsfjorden, Svalbard. Black dots show where tissue samples were taken for RLC, *in-vivo* Chl a emission spectra and pigment analysis. The green algae *Ulva* sp. had dark and non-degraded tissue on the edge of the lamina in January, while in October the tissue was pale green and degraded on the edges of the lamina. *Palmaria palmata* had new tissue growing on the edges of the thallus in January (1) often with a greenish thallus base (2), indicating old tissue with a loss of phycoerythrin (2). In October, the main thallus of *P. palmata* was growing from the thallus of the previous year (3). In the three kelp species, the growth zones are located at the meristem (4) while the older tissue is at the apex of the lamina (5). The sori (S) are also clearly visible for *A. esculenta*, and *L. digitata* in both January and October. (**c**) Sketches show the cycle from the end of the light season in October (left image) to the Polar Night (January, right image) with new tissue in lighter color compared to old tissue (darker). For brown algae, (1y, 2y, 3y) denotes age (in years) of the tissue. The lamina of *S. latissima* during the Polar Night was 40 to 90 cm long. Illustrations in (**c**) by Malin Bø Nevstad.

In October, we collected 3 specimens of each experimental species of macroalgae (*Ulva* sp*., P. palmata, L. digitata, A. esculenta, and S. latissima*) that were kept in an outdoor aquarium with running seawater in the same light and temperature conditions as *in-situ* (+ 1.5 to 2°C, average E_PAR_ of 6.6 μmol m^−2^ s^−1^ measured by the light observatory in October 2020^7^). For each specimen of *Ulva* sp. in January and in October, a subsample of 2–5 cm^2^ of tissue was cut out. For *P. palmata*, tissue subsamples were taken from the base (old tissue) and from the apex of the lamina (new growth). For the 3 kelp species, *A. esculenta, L. digitata,* and *S. latissima*, subsamples were taken from the meristem where new tissue forms on the lamina, and from the apex, which represents the oldest tissue that can be 3–4 years old (Fig. 2)^28^.

### Statistical test

For each subsample collected in January and October (tissue samples from *Ulva* sp., new and old tissue of kelp species) we conducted rapid light curves, Chl a emission spectra and HPLC pigments, giving us triplicate data for each experimental species. One-way ANOVA test was conducted on the data from *Ulva* sp. to see whether there were any significant differences in the E_K_, F_v_/F_m_, and pigment concentrations between seasons. Two-way ANOVA was conducted on the data from the red and brown algae to test for significant differences in the E_K_, F_v_/F_m_, and pigment concentrations between seasons and between tissue age. We report the F-value (representing the variation between sample means versus the variation within the samples) and the *p*-value that determines whether differences between group means are statistically significant. We consider *p* < 0.05 as significant. A post hoc Tukey’s test was conducted to find which variables differed.

### Rapid light curves (RLC)

Rapid light curves (RLC) measure photosynthetic performance of PS II versus irradiance in a similar way to traditional photosynthetic vs irradiance curves (P vs E) but with shorter light incubation periods (here we used 30s) at each irradiance^29^. As a result, steady state conditions of photosynthesis are not achieved in the RLC and the maximum PS II fluorescence quantum yield ($(\phi _{{PSII}}^{{max}}$—more commonly written as F_v_/F_m,_ see Eq. 1) and relative transport rate (rETR) reflect the actual state of photosynthesis (not the optimal state shown by a P vs E curve). RLC reflects the short-term acclimation from the past few minutes but is also influenced by the long-term (weeks to months) acclimation pre-history of algae^29^.

We used the underwater Chl a Fluorometer Diving PAM II from Walz (Effeltrich, Germany) with the WinControl-3 Software (Heinz Walz GmbH, Effeltrich, Germany) for our RLC measurements. All measurements were done either on fully charged battery (9V) or connected to a power supply (12 V). The Diving PAM II uses a red LED with an emission peak of 654 nm as a measuring light source, which we set to E_PAR_ of 0.19 µmol photons m^−2^ s^−1^ for most samples to avoid triggering photosynthetic activity. Samples were exposed to red stepwise increasing E_PAR_ for actinic illumination from 0 to 446 µmol photons m^−2^ s^−1^ in 12 steps (0, 7, 14, 20, 27, 38, 56, 85, 125, 186, 243, 341, and 445 μmol photons m^−2^ s^−1^), with 30s incubation time. The Diving PAM II detected Chl a fluorescence from PS II using a PIN photodiode protected by long-pass and short-pass filters, thus measuring the 730 nm Chl a emission shoulder (termed chl a fluorescence) at 12-bit resolution (dynamic range).

The macroalgae tissue was secured in the magnetic sample holder that was attached to an optical fiber at one end, and the Diving PAM II on the other end. The sample holder was placed in seawater bath without algal tissue at *in-situ* temperature and salinity (-1.8°C in January and + 1.5°C in October) to provide an autozero of Chl a fluorescence prior to measurements on tissues. Once the algal tissue sample was placed in the sample holder, we checked that the fluorescence emission was between 200 and 600 mV at 730 nm to ensure a good signal-to-noise ratio at all irradiances provided. The online fluorescence emission (Ft) was between 200–600 mV and the shape of the fluorescence induction curve was checked to ensure that the minimum fluorescence (F_0_) and the maximum fluorescence (F_m_) were stable before starting the RLC^30^.

We measured the F_0_ and F_m_ after dark acclimation for at least 5 min^30,31^ when approximately all functional reaction centers are open (oxidized state). These also represent the first measurement of the RLC (when E_PAR_ of actinic light = 0). The maximum quantum yield of PS II fluorescence, F_v_/F_m,_ was then calculated on dark acclimated chloroplast following Eq. 1^20,30^.

1 $$ (\phi _{{PSII}}^{{max}}   = F_{v} /F_{m} = \left( {F_{m} - F_{0} } \right)/F_{m}$$

For each E_PAR_ incubation step, we measured the minimum fluorescence in actinic light (F), and the max fluorescence level (F’_m_). The operational PS II fluorescence quantum yield in actinic light ($\phi_{PSII}^{\prime }$) was calculated using the Eq. (2) ^20^

2 $$\phi_{PSII}^{\prime } = \left( {F_{m}{\prime} - F } \right)/F_{m}{\prime}$$

The relative electron transport rate (rETR) is a measure of the photosynthetic rate was calculated from $\phi_{PSII}^{\prime }$ and E_PAR_ (3).

3 $$rETR = \phi_{PSII}^{\prime } *E_{PAR}$$

The RLC data was curve fitted to rETR to find photosynthetic-irradiance parameters based on the Webb et al. (1974) model with the R software v. 3.4.3^32^ using the Phytotools package with the Nelder-Mead fit method Eq. 4 ^33,34^. We obtained the photosynthetic efficiency (α) from the linear part of the RLC and the maximum relative electron transfer rate rETR_max_ (indicating P_max_, maximum photosynthetic rate) to calculate the light saturation parameter, E_K_ (µmol photons m^−2^ s^−1^) Eq. 4–5, ^19,30^.

4 $$rETR = \alpha *E_{K} *(1*e^{{E_{PAR} /E_{K} }})$$

5 $${E_{K} = rETR_{max} /\alpha }$$

### *In-vivo* Chl a emission spectra from PS II

The *in-vivo* Chl a emission spectra under blue and green excitation light show PS II emission peaks at 685 and 730 nm. Blue and green excitation light are absorbed and funneled by light harvesting pigments to Chl a in PS II^17,35,36^. The resulting emission of Chl a fluorescence indicates light energy transfer from light harvesting complexes (LHC II) bonded to in the highly fluorescent PS II (95% of in vivo fluorescence) vs PS I (< 5% of fluorescence)^37–39^. Comparing the relative emission intensities under the different light excitations may help elucidate the relative amount of Chl a bonded to PS II vs PS I.

The algal samples were positioned between a magnetic dark chamber tissue holder and disk (40 mm diameter, 10 mm height) padded with foam rubber. We then used the mini spectrometer on the Diving PAM II, which consists of a polyoxymethylene tube (35 mm diameter, 13 mm height) with blue and green LED excitation bands (light rod). The blue excitation light has a peak at 452 nm, and intensity was set to 200 relative units and green excitation light (peak at 525 nm) intensity set to 600 relative units for each measurement. The spectrometer has an emission spectral range of 400–850 nm with a spectral resolution of 8–10 nm^40^. Each emission spectra were measured in the range 600–850 nm using the auto-range setting that automatically determines the optimal integration time (both excitation and emission). The different integration times are subsequently considered when calculating the units of the light emission in nmol photons m^−2^ s^−1 ^^40^.

### HPLC (high-performance liquid chromatography) isolated light-harvesting pigments

After measurements with Diving PAM II, excess water was wiped off each algal tissue sample, wrapped in aluminum foil and preserved in − 80°C freezer onboard the ship and in the laboratory at the Trondheim Biological Station (TBS) at NTNU, respectively, for further analysis. Samples were kept frozen in dry ice during transport. Each sample was lightly dabbed with paper tissue to remove excess water and then weighed (wet weight). We extracted the chlorophylls and carotenoids in 5 mL of 100% methanol for 4 h in the dark at 4°C (total pigment extraction). We then filtered the pigment extract using a disposable syringe with a 2 µm filter in a 2 mL HPLC vial. The extracts were run through HPLC system from Hewlett Packard 1100 Series, following method described by^41,42^. This HPLC uses an HP 1100 autosampler, HP 1100 series quaternary pump, HP 1100 series thermostatic column compartment and an HP 1100 series diode array detector. The eluent system uses methanol for rinsing, a solution of methanol, acetonitrile, and aquation pyridine (50:25:25) and a solution of acetone and acetonitrile (20:80). Pigments were then detected at 440nm.

### *In-vivo* absorbance spectra

For *P. palmata*, we conducted an additional experiment in May 2022 from the same field site in Kongsfjorden to measure the total *in-vivo* absorbance (dimensionless) of *P. palmata* (all LHC´s and PS´s) and the corresponding fraction of light absorbed by PS II (consisting of LHC II and PS II), denoted a_PSII_^30,39,43^. This is an approach to estimate the fraction of light absorbed by PS I vs PS II enabling us to interpret and verify results from the *in-vivo* Chl a emission spectra. LHC II of the red algae *P. palmata* comprising water soluble phycobiliproteins, dominated by phycoerythrin (PE), bonded to PS II only^21,43^. Since *P. palmata* also has the majority of Chl a bonded to non-fluorescent PSI^21,39,43^ we used data from this species to interpret the *in-vivo* Chl a emission spectra data described in section above.

New growth tissue (apex) of three specimens of *P. palmata* specimens were used. We measured the spectral absorbance of whole tissue and isolated the water soluble fraction of LHC II and PS II between 400 to 800 nm (1 nm spectral resolution, n = 3, see description in next paragraph). We used a QE Pro spectrometer (Ocean Insight Inc., Orlando, FL, USA) equipped with a 1 cm cuvette fitted into an Ocean Insight cuvette holder. The light-leading optical fiber (Ocean Optics QP1000-2-VIS-BX VIS/NIR) was pointing at the cuvette at one end and a corresponding optical fiber (Ocean Optics QP1000-2-VIS-BX VIS/NIR, 2 m long and 1 mm in fiber diameter) measured the absorbance on the other end of the cuvette in a dark room. The light source was an HL-2000-HP high-power tungsten halogen light source from Ocean Insight Inc. (Ocean Insight Inc., Orlando, FL, USA).

We used filtered seawater (with no algae sample) as a blank. For the total *in-vivo* absorbance spectra, a section of the lamina was put into a 1 × 1 cm (4 mL) quartz cuvette with 1 cm optical path length. The tissue was pressed vertically onto the detector side of the cuvette, with the detection sensor directed at the light source (transmittance/absorbance set up) and with an integration time of 100 ms. After measurement of the tissue, the sample was taken out of the cuvette and grinded with a porcelain mortar and pestle with ca. 20 ml filtered seawater (0.1 µm filter) at *in-situ* temperature (used as a buffer and extractant[ 21, 43]). The mortar and pestle were used for 10 min to extract the water-soluble light-harvesting pigments, i.e., the phycobiliproteins dominated by PE (LHC II and PS II) found in *P. palmata*^43^. The pigment protein extract was then filtered using a 10 mL syringe with 0.4 µm polycarbonate filter and transferred to a quartz cuvette for spectral absorbance measurements. The filtered solution, free of light-scattering particles, was pink and clear (no scattered light detected).

The absorbance spectra were scaled to an absorbance of 0 at 800 nm (light scattering correction). After this, the spectra were normalized so that the PE peaks at 495 and 545 nm of LHC II and PS II spectra matched the corresponding peaks of the in vivo absorption spectra. A match at 495 nm indicates that 100% of the phycobiliproteins (LHC II) are bonded to PS II^39,43^. The difference spectra between the total absorbance curve and the fraction of light absorbed by a_PSII_ represents the non-fluorescent fraction of LHC I and PS I was done in accordance with Johnsen and Sakshaug^37^.

### *In-vivo* reflectance spectra

Spectral reflectance is essential for mapping method that involve hyperspectral imaging and can be used to study biodiversity and biomass of macroalgae [examples 25, 26]. The reflectance spectra are determined by pigment-specific bio-optical properties of algae.

Reflectance measurement of green (*Ulva* sp.), red (*P. palmata*), and brown algae (*A. esculenta, L. digitata, and S. latissima*) were conducted in January (described in Summers et al. 2020^25^) using the QE Pro spectrometer with the same type of light source and fibers as described above. The same method was used in May to measure reflectance on the green algae *Ulva* sp., the red algae *P. palmata* and the brown algae *S. latissima*. For reflectance measurements, the light source and detection fiber were at a 45° angle from the algal tissue.

## Results

### Morphology

Macroalgal specimens collected in January 2020 were, in general, in better health condition indicated by visual inspections than the same species collected in October 2020 (Fig. 2). In the case of *Ulva* sp. (Chlorophyte), specimens were visually darker green in January and with no partial degradation at the edges of the lamina compared to the specimens collected in October. In January, the new tissue of *P. palmata* (Rhodophyte) was growing at the apex of the lamina and around the edges of the old part of the lamina. In contrast, in October, the new tissue was growing at the apex of the lamina. The lamina of the brown algal (Phaeophytes) kelp species (*A. esculenta, L. digitata* and *S. latissima*) were characterized by 3-year-old tissue at the apex of the lamina, 2-year-old tissue in the mid-section and tissue of 1-year-old or less at the meristem (new growth, Fig. 2). Fertile tissue (sorus) could be detected on all kelp species in both seasons except for *S. latissima* in January. The 2- and 3-year-old part of the lamina of the kelp species showed signs of deterioration. In the cases of the *A. esculenta* and *S. latissima*, the lamina was shorter (reduced and partly degenerated) in October compared to January.

### Rapid light curves (RLC)

**Figure 3:**
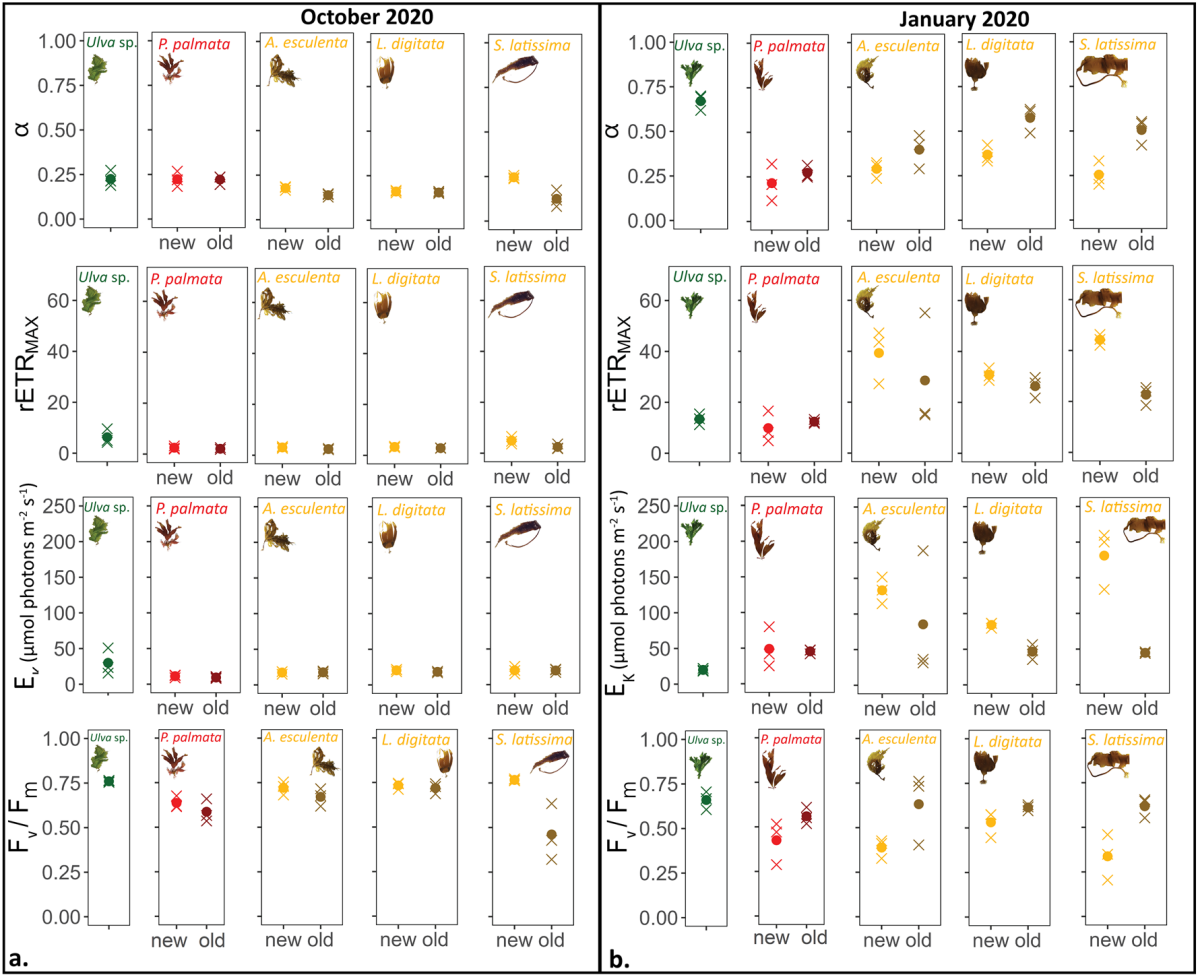
Photosynthetic parameters in green, red, and brown macroalgae, comprising photosynthetic efficiency (α), maximum relative electron transport rate (rETR_max_), light saturation parameter (E_K_), and the maximum quantum yield in the dark (F_v_/F_m_) derived from RLC measured in (**a**) October and (**b**) January. The mean values are represented by dots and the individual measurements (n = 3) are represented by crosses. For statistical significance see RLC section in results.

Overall, the photosynthetic parameters differed significantly between seasons in all species. The E_K_ was significantly different between January and October for red and brown algae. There were also significant differences between new tissue and old tissue in January as detailed below. (Fig. 3, Table 1).

**Table 1:** ANOVA comparing the effects of seasons and tissue age on the photosynthetic parameters in green, red, and brown algae.

Species	Effects	PS II parameters	F value	*p*
*Ulva* sp.	Seasons	α	**149.2**	***p* < 0.001**
		**rETR**_**max**_	**11.78**	***p* < 0.01**
		E_k_	0.858	*p* > 0.05
		**F**_**v**_**/F**_**m**_	**11.63**	***p* < 0.01**
*P. palmata*	Seasons	α	0.39	*p* > 0.05
		**rETR**_**max**_	**24.66**	***p* < 0.001**
		**E**_**k**_	**20.289**	***p* < 0.001**
		**F**_**v**_**/F**_**m**_	**7.71**	***p* < 0.01**
	Tissue age	α	0.65	*p* > 0.05
		rETR_max_	0.36	*p* > 0.05
		E_k_	0.10	*p* > 0.05
		F_v_/F_m_	0.98	*p* > 0.05
*A. esculenta*	Seasons	α	**36.12**	***p* < 0.001**
		**rETR**_**max**_	**18.21**	***p* < 0.001**
		**E**_**k**_	**12.11**	***p* < 0.001**
		**F**_**v**_**/F**_**m**_	**9.01**	***p* < 0.01**
	Tissue age	α	1.19	*p* > 0.05
		rETR_max_	0.61	*p* > 0.05
		E_k_	0.81	*p* > 0.05
		F_v_/F_m_	2.42	*p* > 0.05
*L. digitata*	Seasons	α	**150.00**	***p* < 0.001**
		**rETR**_**max**_	**328.29**	***p* < 0.001**
		**E**_**k**_	**193.49**	***p* < 0.001**
		**F**_**v**_**/F**_**m**_	**39.37**	***p* < 0.001**
	Tissue age	α	**15.39**	***p* < 0.001**
		rETR_max_	3.03	*p* > 0.05
		**E**_**k**_	**36.00**	***p* < 0.001**
		F_v_/F_m_	2.01	*p* > 0.05
*S. latissima*	Seasons	α	**36.65**	***p* < 0.001**
		**rETR**_**max**_	**491.81**	***p* < 0.001**
		**E**_**k**_	**59.33**	***p* < 0.001**
		**F**_**v**_**/F**_**m**_	**4.95**	***p* < 0.05**
	Tissue age	α	4.07	*p* > 0.05
		**rETR**_**max**_	**80.69**	***p* < 0.001**
		**E**_**k**_	**32.05**	***p* < 0.001**
		F_v_/F_m_	0.04	*p* > 0.05

Significant values (*p* < 0.05) are shown in bold.

For the green algae *Ulva* sp., F_v_/F_m_ was significantly lower in January versus October (*F* = 11.63, *p* > 0.01). α and rETR_max_ were both significantly higher in January then in October (*F* = 149.2, *p* < 0.001 for α and *F* = 11.78, *p* < 0.01 for rETR_max_). However, E_K_ did not change significantly (*p* > 0.05) between seasons.

For the red algae, *P. palmata*, F_v_/F_m_ differed significantly between seasons (*F* = 7.71, *p* < 0.01) with F_v_/F_m_ being lower in new tissue in January versus new tissue in October (Tukey post-poc test). There was not a significant difference of the photosynthetic parameters between old and new tissue (α of 0.25–0.30 and rETR_max_ of ~ 15 giving E_K_ of ~ 50 µmol photons m^−2^ s^−1^, Fig. 3). However, there was a significant difference in the E_K_ between seasons (*F* = 20.289, *p* < 0.001 with higher E_K_ in new tissue in January compared to both new and old tissue in October (Tukey post-hoc test).

In the case of the kelp species, seasons significantly affected F_v_/F_m_ as well as E_K_ in all 3 species, and tissue age was significant factor for E_K_ in *L. digitata* and *S. latissima*. New tissue of *A. esculenta, L. digitata and S. latissima* had lower F_v_/F_m_ in January than in October (*F* = 9.01, *p* < 0.01,* F* = 39.373, *p* < 0.001, *F* = 4.954, *p* < 0.05 respectively). For S. *latissima*, F_v_/F_m_ was also significantly lower in new tissue compared to old tissue for both January and October. E_K_ in new tissues in January were generally two times higher E_K_ (100–200 µmol photons m^−2^ s^−1^) than the old tissues in January (E_K_ ~ 50 µmol photons m^−2^ s^−1^). In contrast, in October, there was no significant difference of the photosynthetic parameters between old and new tissues (α of 0.25 and rETR_max_ < 15 resulting in E_K_ < 50 µmol photons m^−2^ s^−1^). Specifically, for *A. esculenta*, E_K_ was significantly different between seasons (*F* = 12.106, *p* < 0.001) with the Tukey post-hoc test showing that E_K_ was higher in new tissue in January compared to new and old tissue in October. For *L. digitata*, E_K_ differed significantly between seasons (*F* = 48.873, *p* < 0.001) and between tissue age (*F* = 5.605, *p* < 0.01). E_K_ of both new tissue and old tissue from January were higher than new and old tissue from October, respectively. In addition, during the Polar Night, E_K_ was higher in new tissue than in old tissue. Similarly, E_K_ in *S. latissima* was significantly different between seasons (*F* = 59.33, *p* < 0.001) and between tissue age (*F* = 32.05, *p* < 0.001). E_K_ for new tissue was higher in January than in October. In addition, E_K_ in January was higher in new tissue compared to old tissue.

### Pigments

**Figure 4:**
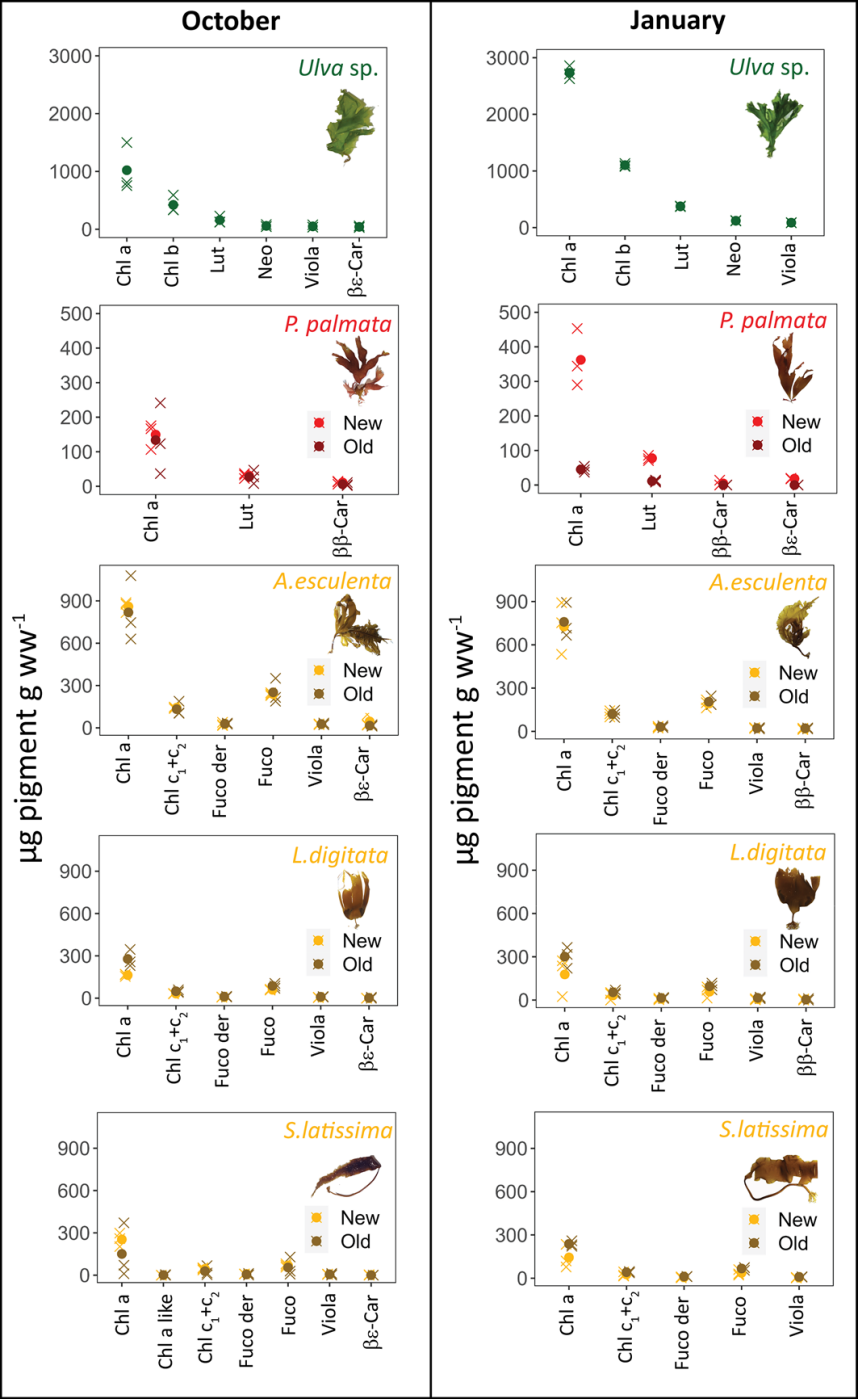
Pigment concentrations (µg pigment g ww^−1^) in green, red, and brown macroalgae in new tissue (left box in light brown or red) and old tissue (right box in dark brown or red), as well as between seasons (left: October, right: January). The mean values are represented by dots and the individual measurements (n = 3) are represented by crosses. Pigment nomenclature from^42^ with pigment abbreviations as follows: Chl a = chlorophyll a, Chl b = chlorophyll b, Lut = lutein, Neo = neoxanthin, Viola = violaxanthin βε-Car =  βε carotene, ββ-Car =  ββ carotene, Chl c_1_ + c_2_ = chlorophyll c_1_ and c_2_, Fuco = fucoxanthin, Fuco der = fucoxanthin derivative. For statistical significance see Pigments section in results.

Figure 4 shows the differences in pigment concentrations between January and October as well as between new and old tissue with the results of the Anova tests in Table 2. Chlorophyll a was the dominant pigment in all species (Fig. 4). In *Ulva* sp., concentration of light harvesting pigments per wet weight were significantly higher (at least twice as high) in January compared to in October for all major pigments. Chlorophyll a was ~ 2500 µg Chl a g ww^−1^ in January compared to ~ 1000 µg Chl a g ww^−1^ in October (*F* = 47.19, *p* < 0.001), chlorophyll b (Chl b) concentrations were ~ 1000 µg Chl b g ww^−1^ in January vs ~ 400 µg Chl b g ww^−1^ in October (*F* = 62.4, *p* < 0.001), Lutein (Lut) were ~ 350 µg Lut g ww^−1^ in January vs ~ 150 µg Lut g ww^−1^ in October (*F* = 38.26, *p* < 0.001), followed by neoxanthin (neo) and violaxanthin (viola) with ~ 80 and ~ 112 µg pigment g ww^−1^ respectively in January and ~ 40 and ~ 50 µg pigment g ww^−1^ respectively in October (*F* = 27.04, *p* < 0.001 for neo and *F* = 28.04, *p* < 0.001 for viola). Additionally, βε carotene (βε car) was found only in October (~ 50 µg βε-car g ww^−1^).

**Table 2:** ANOVA comparing the effects of seasons and tissue age on the pigment concentrations in green, red, and brown algae.

Species	Effects	Pigments	F value	*p*
*Ulva* sp.	Seasons	**Chl a**	**47.19**	***p* < 0.001**
		**Chl b**	**62.4**	***p* < 0.001**
		**Lut**	**38.26**	***p* < 0.001**
		**Neo**	**27.04**	***p* < 0.001**
		**Viola**	**28.04**	***p* < 0.001**
*P. palmata*	Seasons	Chl a	2.45	*p* > 0.05
		**Lut**	**5.69**	***p* < 0.01**
		ββ-Car	4.18	*p* > 0.05
	Tissue age	**Chl a**	**17.55**	***p* < 0.001**
		Lut	59.00	***p* < 0.001**
		ββ-Car	2.71	*p* > 0.05
*A. esculenta*	Seasons	Chl a	1.10	*p* > 0.05
		Chl c	0.91	*p* > 0.05
		Fuco	2.60	*p* > 0.05
		Viola	1.82	*p* > 0.05
	Tissue age	Chl a	0.003	*p* > 0.05
		Chl c	0.10	*p* > 0.05
		Fuco	0.20	*p* > 0.05
		Viola	0.24	*p* > 0.05
*L. digitata*	Seasons	Chl a	1.01	*p* > 0.05
		Chl c	0.05	*p* > 0.05
		Fuco	0.06	*p* > 0.05
		Viola	2.35	*p* > 0.05
	Tissue age	Chl a	0.003	*p* > 0.05
		Chl c	4.18	*p* > 0.05
		Fuco	5.22	*p* > 0.05
		**Viola**	**9.65**	***p* < 0.01**
*S. latissima*	Seasons	Chl a	0.03	*p* > 0.05
		Chl c	0.06	*p* > 0.05
		Fuco	0.22	*p* > 0.05
		Viola	0.03	*p* > 0.05
	Tissue age	Chl a	0.01	*p* > 0.05
		Chl c	0.003	*p* > 0.05
		Fuco	0.16	*p* > 0.05
		Viola	1.46	*p* > 0.05

Significant values (*p* < 0.05) are shown in bold.

In new tissue of *P. palmata* there was significant difference in Chl a concentrations for tissue age (*F* = 17.553, *p* < 0.001) with 2 × higher concentrations of Chl a in new tissue in January (~ 350 µg Chl a g ww^−1^) than in new tissue in October (~ 150 µg Chl a g ww^−1^) and old tissue from January (~ 45 µg Chl a g ww^−1^). Lutein concentrations per wet weight followed a similar trend with significant differences over seasons (*F* = 5.689, *p* < 0.01) and tissue age (*F* = 29.003, *p* < 0.001). Lutein was found in higher concentration in new tissue in January (~ 75 µg Lut g ww^−1^) compared to new tissue in October (~ 30 µg Lut g ww^−1^) and old tissue in January (~ 10 µg Lut g ww^−1^). ββ carotene (ββ car) and βε carotene (βε car) were found only in the new tissue in January (~ 4 µg pigment g ww^−1^ and ~ 20 µg pigment g ww^−1^ respectively). ββ carotene was found in both new and old tissue in October (~ 10 and ~ 5 µg ββ-car g ww^−1^ respectively).

Concentration of pigments per wet weight in brown algae were highest in *A. esculenta*, followed by, *L. digitata* and *S. latissima*. Only slight differences (but not significant, *p* > 0.05) in pigment concentrations were observed between January and October, whereas there were some stronger differences (but not significant, *p* > 0.05) in pigment concentration between new and old tissue. *Alaria esculenta* had similar Chl a concentration in both new and old tissue at ~ 750 µg Chl a g ww^−1^ in January and 820–860 µg Chl a g ww^−1^ in October. *L. digitata* had 175 and 300 µg Chl a g ww^−1^ in new and old tissue respectively in January compared to 160 and 275 µg Chl a g ww^−1^ in new and old tissue in October. *Saccharina latissima* had ~ 130 and 300 µg Chl a g ww^−1^ in new and old tissue respectively in January compared to 340 and 162 µg Chl a g ww^−1^ in new and old tissue in October. In most of the brown algae older tissue had slightly more Chl a than the new tissue by a factor of 1.5 to 2, except for *A. esculenta* that was similar in both seasons, and *S. latissima,* where new tissue had 2 times more Chl a than old tissue in October.

Concentrations of Chl c (Chl c_1_ + c_2_), which is a characteristic pigment in brown algae, were similar between seasons, with variations in concentrations between new and old tissue in some species. *Alaria esculenta* had similar concentrations of Chl c across all measurements with ~ 120–140 µg Chl c g ww^−1^. *Laminaria digitata* had higher concentrations of Chl c in old tissue with 50 µg Chl c g ww^−1^ compared to new tissue (30 µg Chl c g ww^−1^) in both seasons. In contrast, *S. latissima*, in January, had lower pigment concentration in the old tissue with ~ 23 µg Chl c g ww^−1^ compared to new tissue ~ 45 µg Chl c g ww^−1^. However, the reverse was seen in *S. latissima* in October with new tissue having higher concentrations of Chl c (~ 60 µg Chl c g ww^−1^) compared to old tissue (~ 30 µg Chl c g ww^−1^).

Fucoxanthin (fuco) was the second most abundant light harvesting pigment in the brown algae species. Fucoxanthin was found in slightly lower concentrations in *A. esculenta* in January compared to October (~ 195–205 µg fuco g ww^−1^ in January vs ~ 240–250 µg fuco g ww^−1^ in October). Similarly, *L. digitata* had lower concentrations of fuco in new tissue (~ 60 µg fuco g ww^−1^ in both seasons) compared to old tissue (~ 85–95 µg fuco g ww^−1^). Concentrations of fuco in *S. latissima* was lower in the new tissue in January (~ 30 µg fuco g ww^−1^) compared to old tissue in January (~ 75 µg fuco g ww^−1^). However, in October, new tissue had higher concentrations of fuco (~ 100 µg fuco g ww^−1^) compared to older tissue (~ 60 µg fuco g ww^−1^). Violaxanthin were found in lower concentrations but followed a similar trend to the other pigments in the brown algae. *Alaria esculenta* had ~ 22 and ~ 25 µg viola g ww^−1^ in January and October respectively with no difference between new and old tissue (*p* > 0.05). Similarly, old tissue of *L. digitata* had 2 × the viola concentration of the old tissue in both seasons (~ 5 µg viola g ww^−1^ in new tissue vs ~ 10–15 µg viola g ww^−1^ in old tissue). Whereas, in January, new tissue of *S. latissima* had a concentration of 5 µg viola g ww^−1^ compared to ~ 5 µg viola g ww^−1^ in old tissue. In October, the new tissue of *S. latissima* had 10 µg viola g ww^−1^ compared to old tissue which had ~ 5 µg viola g ww^−1^. Finally, in January, in *S. latissima*, we found ββ car with concentrations of ~ 3–11 µg ββ car g ww^−1^. In contrast, in October we found βε car with concentrations of 3–45 µg βε car g ww^−1^.

### *In-vivo* Chl a emission spectra from PS II under blue and green excitation light

**Figure 5:**
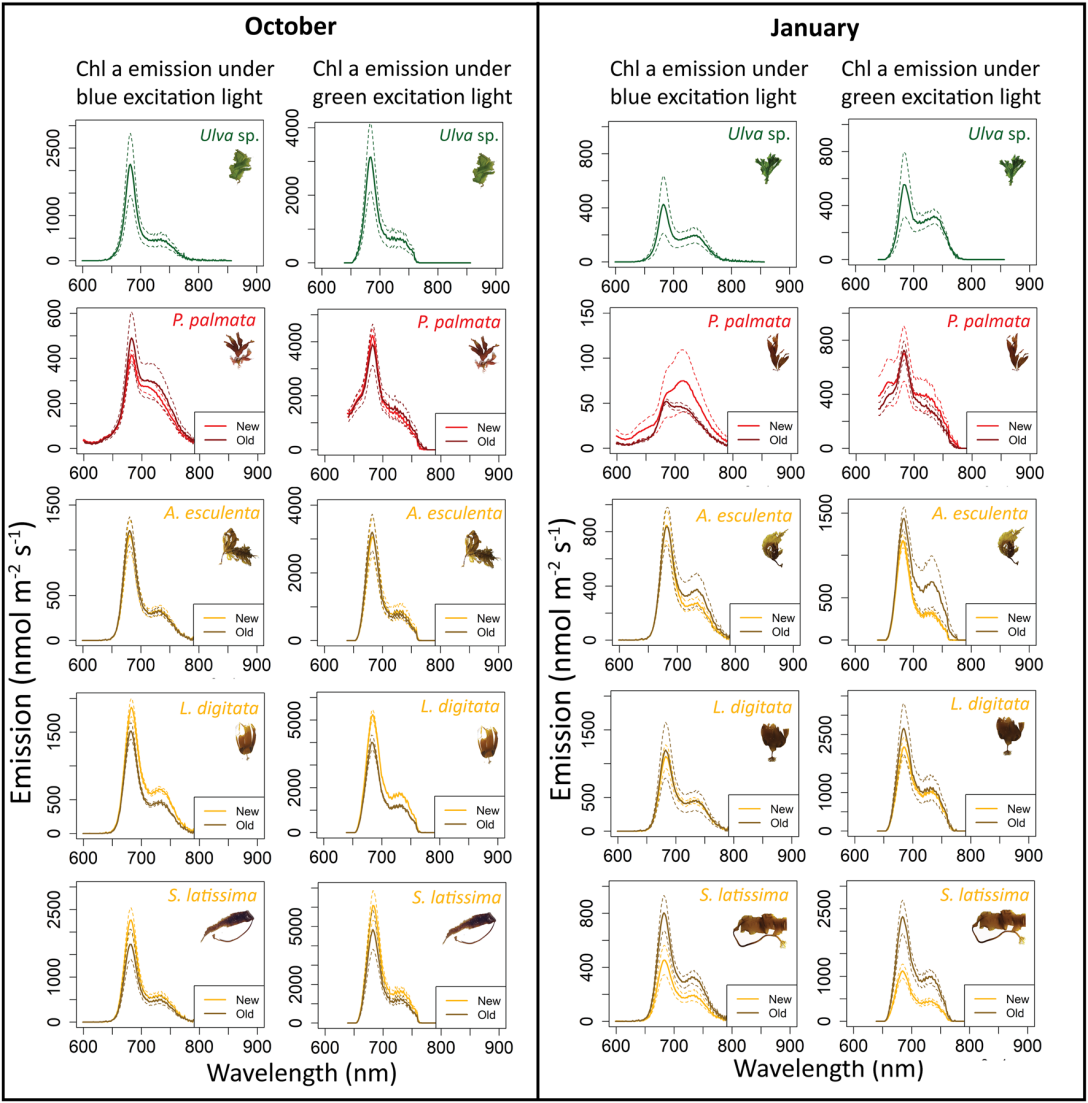
In-vivo Chl a emission spectra from PS II under blue and green excitation light for green, red, and brown algae, measured in October (left) and in January (right). Solid line represents the mean ( n = 3) and the dotted line is the standard deviation.

The *in-vivo* Chl a emission spectra represent the fluorescence emission of Chl a from PS II characterized by an emission peak at 685 nm and a corresponding shoulder at 730 nm (Fig. 5). Most emission spectra had the same shape except for January spectra of *P. palmata* that had no peak at 685 and a reduced shoulder at 730 under blue excitation light. However, the emission intensity varied between species and seasons.

For October samples, the Chl a fluorescence emission spectra followed a similar trend as the January emission spectra for all experimental species. However, the emission intensity was ~ 4 × higher in October compared to January in *Ulva sp*. and *P. palmata* and ~ 2 × higher for the kelp species .

In *Ulva* sp., Chl a fluorescence emission was slightly higher under green excitation light relative to tissue exposed to blue excitation light (fluorescence emission mean of ~ 600 vs ~ 400 nmol m^−2^ s^−1^ in January and ~ 3000 vs ~ 2500 nmol photons m^−2^ s^−1^ in October). In contrast, *P. palmata*, Chl a emission was ~ 10 × higher when exposed to green excitation light relative to blue excitation light (fluorescence emission mean of ~ 750 vs ~ 75 nmol photons m^−2^ s^−1^ in January, ~ 4000 vs ~ 500 nmol photons m^−2^ s^−1^ in October). In addition, there was more variability in the new tissue than in the older tissue. Similarly, in brown algae, Chl a emission was ~ 2 × higher under green excitation light.

### *In-vivo* absorbance and reflectance spectra

**Figure 6:**
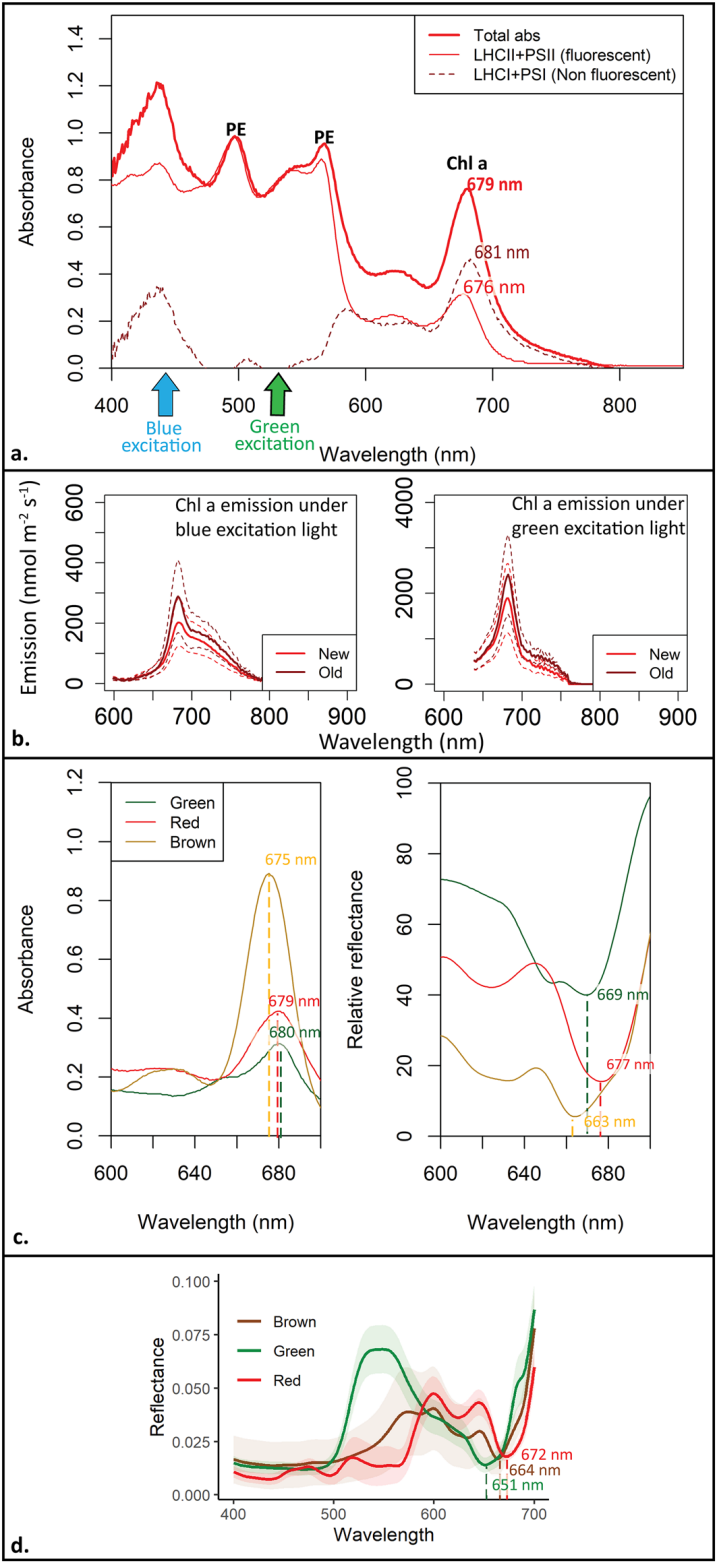
(**a**) Normalized spectral absorbance measurements for phycobiliprotein-containing red alga *Palmaria palmata*. Thick red solid line: total *in-vivo* pigment absorbance spectra, thin red solid line: absorbance of light harvesting complex II (LHC II) (mainly phycoerythrin, PE) and PS II that provides 95% of Chl a fluorescence signal. Dark red dashed line: Absorbance of light harvesting complex I (LHC I) and PS I (non-fluorescent). The phycobiliprotein peak, dominated by PE, is at 495 nm with a shoulder at 545–560 nm. The Chl a absorbance peaks at 679 nm and 681 nm indicates that the majority of Chl a is bonded to PS I, and the Chl a peak at 676 nm indicates Chl a bonded to PS II, highlighting a spectral shift. Blue and green arrows show the excitation light peaks (452 nm, 525 nm) used for Chl a emission spectra measurements. (**b**) emission spectra of *P. palmata* under blue (left) and green (right) excitation light. Light red solid line represents new tissue and dark red solid line represents old tissue with the dotted lines representing standard deviation. (**c**) *In-vivo* spectral absorbance and reflectance of whole tissue of green (*Ulva* sp.), red (*P. palmata*), and brown (*S. latissima*) algae, showing spectral shift of Chl a bonded to PS I or PS II. (**d**) In-vivo reflectance spectra from the Polar Night (January 2020, detailed in Summers et al. ^26^) of green (*Ulva* sp.), red (*P. palmata*) and brown algae (average of *A. esculenta, L. digitata, and S. latissima*). Light colors indicate the standard error.

The *in-vivo* absorbance curves of *P. palmata* (Fig. 6a) show PE peaks at 540 nm and 570 nm for the total absorbance and the corresponding fraction of light absorbed by a_PSII_. The *in-vivo* Chl a absorbance red peak was at 679 nm for the total absorption of *P. palmata* and at 676 nm for fraction of light absorbed by a_PSII_. The Chl a peak in the non-fluorescent fraction of LHC I and PS I was at 681 nm. We, thus, observed a shift of 5 nm in the Chl a absorbance peak between the fluorescent (LCH II and PS II) and non-fluorescent (LHCI and PSI) spectra. In addition, the Chl a peak of fraction of light absorbed by a_PSII_ was 40% of the total absorption.

The *in-vivo* emission spectra of *P. palmata* under blue and green excitation light measured in May (Fig. 6b) was similar to the emission spectra of the same species measured in October (Fig. 5) characterized by an *in-vivo* emission peak at 685 nm and a corresponding shoulder at 730 nm. Chlorophyll a emission was ~ 10 × higher when exposed to green excitation light relative to blue excitation light (fluorescence emission mean of ~ 200–300 nmol photons m^−2^ s^−1^ versus, ~ 2000–2500 nmol photons m^−2^ s^−1^) with some variation between new and old tissue.

The total *in-vivo* absorbance of green, red, and brown algae measured in May showed a spectral shift in the Chl a peak which was at 680 nm, 679 nm and 675 nm respectively (Fig. 6c). Similarly, Chl a dip in the reflectance spectra from May was observed at 669 nm for green algae, 677 nm for red algae and 663 nm for brown algae. In January, the Chl a dip in the reflectance curve was observed at 651 nm for green algae, 672 nm for red algae and 664 nm for brown algae (Fig. 6d) which differed from the May measurements (Fig. 6c). No wavelength shifts in the red absorbance peak and corresponding reflectance dip were observed between the triplicates of the same species in May.

## Discussion

Our results show that the macroalgae species studied were in better physical state during the Polar Night (in January) than at the end of the light season (in October). Visual evidence for this was the physical appearance of the macroalgal specimens during Polar Night (Fig. 2). In addition, new tissue growth was observed on *P. palmata,* as well as longer lamina in *S. latissima* and *A. esculenta*, providing strong evidence that these species of macroalgae were forming new tissue during the Polar Night. Other species, *Ulva* sp., and *L. digitata,* also grew new tissue during the Polar Night but to a lower extent than *P. palmata, A. esculenta,* and *S. latissima.* Dissolved nutrient concentrations (such as nitrate) in Svalbard are high in winter with concentrations of 10–12 µmol L^−1 ^^9^ and close to depleted in summer due to phytoplankton blooms^2,8,44^. Kelp species are able to store carbohydrates in the form of polymer laminarins (also called laminaran, a storage of glucan product of photosynthesis) in their lamina and utilize them to maintain basic physiological functions during winter^9^. However, we suggest that macroalgae during the Polar Night are likely utilizing the available nutrients together with their own carbohydrate storage, not only to maintain photophysiological fitness (discussed below) but to grow new tissue. Growth during the winter (dark season) has been reported in Antarctica, where some red algae species grow new tissue^8^. In contrast*,* at the end of the light season, the lamina of the kelp species were at their peak state of erosion with seasonal high in epi-growth and the oldest upper parts (~ 3-year-old tissue) disintegrating in accordance with previous findings during the Polar Night^2^. Meltwater from surrounding glaciers and river-run off peak in July–August^45^ causes a reduction in the underwater light intensity^46^ and disintegration of the kelp lamina.

During the Polar Night, the photosynthetic apparatus of macroalgae studied were functioning as demonstrated by the presented photosynthetic parameters (Fig. 3). Both green algal tissue and new tissue from red algae had lower F_v_/F_m_ in January compared to October. *Palmaria palmata* also had higher E_K_ in all tissue during the Polar Night. In the kelp species, new tissue in the Polar Night had lower F_v_/F_m_ and higher E_K_ than at the end of the light season. At the end of the light season, kelp species are acclimated to low light similar to macroalgae living in low light environments. For example, algae living at depth lower than 10m are characterized by an E_K_ of ~ 40 μmol photons m^−2^ s^−1^ reported in^47–49^. Thus, high values of E_K_ here cannot be interpreted as high light acclimation, but as an indicator of a slow working PS II. Similarly, lower F_v_/F_m_ is usually interpreted as a sign of stress as it indicates excess energy dissipation as heat in the antenna complex, resulting in a decreased quantum yield^50^. Our results of lower F_v_/F_m_ during the Polar night, confirm that although the algae are active, they also have a slower functioning PS II. In the case of *S. latissima*, we observed significantly lower F_v_/F_m_ in new tissue versus old tissue at the end of the light season, which could also indicate a separation of function between different sections of the lamina. Such is the case in the large kelp species, *Macrocystis pyrifera* that grows up to 30 m in length*,* where the tissue close to the meristem capitalizes on nutrients, whereas the apex of the lamina (old tissue) focuses more on photosynthesis^4,51^.

The light harvesting pigments in the macroalgae studied were non-degraded and functional during both seasons as shown by the shape of the *in-vivo* Chl a emission spectra of PS II characterized by a peak at 685 nm and a corresponding shoulder at 730 nm^52^. Light harvesting pigments play an important role in photophysiology of macroalgae as they capture photons and transfer them to the reaction centers^17,35,36^. Examining them is important to understand the relationship between the macroalgae and the environment they live in, especially in regards to the quality of light (light intensity and wavelength availability) (21, 43). In green algae, Chl a, Chl b and lut are the main light-harvesting pigments that absorb blue and red light (Fig. 4). The main light-harvesting pigments in the red algae *P. palmata* are phycobiliproteins (dominated by PE), which absorb light in the blue-green to green part of the spectrum (Fig. 6)^2^ and then funnel the light energy to Chl a in PS II^21^. In brown algae, the main light-harvesting pigments are Chl a, Chl c and fuco (Fig. 5), where fuco also absorbs in the blue-green (460–535 nm). In brown algae, fuco is also the dominating light-harvesting pigment bonded to PS II^2,43^. In addition to their role in photosynthesis, pigment composition determines which wavelengths of light available can be harvested. As mentioned above, water clarity varies between seasons, with phytoplankton blooms in spring and highest sedimentation from melt water and river run-off in July–August^9^. Thus, even though light availability in air is high from May to October, the corresponding underwater light availability can be highly reduced due to phytoplankton, suspended matter, and colored dissolved organic matter.

We observed that *Ulva* sp. and *P. palmata* accumulated pigments during the Polar Night. Pigment accumulation under low light conditions (such as under ice) were found to be correlated with an increase in maximum photosynthetic rates in green, red, and brown algae from Kongsfjorden^53^, indicating that the algae were acclimatizing to decreasing light. In contrast, in kelp species, we observed stable pigment concentrations between seasons. Thus, they were not acclimating to the light conditions of the Polar Night by regulating pigment concentrations. Therefore, the reduced photosynthetic activity must be due to PS II not being fully active in the kelp species examined. We hypothesize that there may be a shortage or a slow turnover of the D1 protein during the Polar Night. The polypeptide D1 is crucial to the electron transfer as they bind Chl a for optimal photosynthesis^54^. However, the synthesis of the D1 protein is activated by light at a given pH optimum and the absorbed quanta is dependent on a functional photosynthetic unit comprising of LHC II, LHC I, PS II, and PS I^55^. The D1 protein synthesis and activation during the Polar Night and as light becomes available may be a major component to be studied in the future.

The pigment distribution between PS I and PS II is an important factor altering the Chl a emission spectra. The corresponding *in-vivo* absorbance and a_PSII_ in *P. palmata* enable us to determine the fraction of Chl a in PS I and II. We use *P. palmata* in May as a case study to illustrate how the absorbance spectra helped us interpret the emission spectra (Figs. 5 and 6). Measurements from *P. palmata* show that, the emission of Chl a in PS II under green excitation light was 8 to 10 × higher than under blue excitation light where Chl a in PSI is excited (Fig. 6b), in accordance with Grzymski et al.^43^. When adjusting for differences in the excitation light intensity settings of PAM-spectrometer, the signal from the green emission needs to be decreased by 33% more than what is shown in Figs. 5 and 6b to provide the same energy output in blue and green. If this is adjusted for, the emission of Chl a in PS II was 3 × higher (in May) under green excitation light than under the blue excitation light. We suggest that the difference in the intensity of the emission spectra under green (PS II) and blue (PS I) excitation light is an indication of differences in light energy transfer to PS II relative to PS I with 30% of Chl a in PS II and the rest in PS I. This is comparable to the results of using the spectral absorbance.

By using a_PSII_, outlined in Fig. 6a, we calculated the fraction of Chl a bonded to PS II. The Chl a peak at 676 nm a_PSII_ was ~ 40% of the Chl a peak at 679 nm of the *in-vivo* total absorbance. We, therefore, conclude that ~ 40% of Chl a in *P. palmata* is bonded to PS II with most of the Chl a (60%) bonded to PS I. This is in accordance with both Grzymski et al.^43^ and Johnsen and Sakshaug^39^. We, thus, observed a slight difference in estimated [Chl a] in PS II of 10% between the interpretated *in-vivo* Chl a emission spectra and the a_PSII_ calculated fraction (Fig. 6).

Our Chl a emission data for green, red, and brown macroalgae indicate that there are variations between species, tissue age, and seasonality (Fig. 5). However, further investigation with different intensity settings of the spectrometer excitation light is needed to align the emission spectra interpretation with the a_PSII_ results. Calculating the fraction of Chl a in PS II vs PS I is necessary to accurately calculate O_2_ production from PAM fluorescence measurements^37^. A factor of 0.5 or 0.6 (50 and 60% of Chlorophyll a in PS II) is usually used by default to calculate the theoretical fraction of quanta absorbed^37,39,52^. This is the first study, to our knowledge, using the mini spectrometer of the Diving PAM II to compare Chl a emission spectra between species and seasons, additional challenges to the interpretation may be caused by the package effect (intracellular self-shading) that includes differences in the tissue thickness, pigment concentration in the chloroplasts, density of chloroplast and non-photochemical quenching^43^. More research looking at the Chl a emission spectra from different pigment groups of algae, grown under different environmental conditions, and with different intensity and integration time settings of Chl a emission spectrometer, is needed to investigate the package effect as well as to validate our interpretations and assumptions.

We observed a spectral shift of 3 nm in the *in-vivo* absorbance peak of Chl a relative to the a_PSII_ in the red absorption peak region. The Chl a absorbance peak was at 679 nm for the total absorbance, at 676 nm for a_PSII_, and at 681 nm indicating that the majority of Chl a is attached to PS I. This difference spectrum is in accordance with our findings discussed above (Fig. 6a). The observed spectral shift is due to Chl a being bonded to different proteins in PS I, PS II and their corresponding light-harvesting complexes (LHC I and II)^38,39^. With most of the Chl a in *P. palmata* bonded to PS I, the fluorescence emission detected by the Diving PAM II was low both for PS II kinetics (fluorescence yield measurements) and corresponding *in-vivo* Chl a emission spectra.

We also observed a spectral shift in the *in-vivo* Chl a (644–680 nm), Chl b (650 nm), and Chl c (600 and 630 nm) peaks in the absorbance and corresponding reflectance spectra of green, red, and brown algae indicating the fraction of Chl a bonded to PS II and PS I as well as their corresponding light-harvesting complexes (Fig. 6b,c). The *in-vivo* Chl a red absorbance maximum peak in brown algae (*S. latissima*) was at 675 nm, indicating that most of the Chl a is bonded to PS II. In contrast, green (*Ulva* sp.) and red (*P. palmata*) algae have the *in-vivo* Chl a absorbance peak at 680 nm and 679 nm, respectively, indicating that the majority of Chl a is bonded to PS I in accordance with our interpretation of the emission spectra detailed above. These results are in accordance with phytoplankton classes of chromophytes (Chl c containing phytoplankton classes), Chl b containing phytoplankton (chlorophytes, euglenophytes, and prasinophytes), and phycobiliprotein containing cryptophytes and cyanobacteria, outlined in Johnsen and Sakshaug^39^. Similarly, we observed a shift in the *in-vivo* spectral reflectance dips in the red part of the spectrum. The reflectance dip in green algae was lower at 651 nm in January and 669 nm in May probably caused by the relatively high absorbance of Chl b (650 nm) and Chl a attached to PS I (679–680)^43^. In contrast, the *in-vivo* reflectance dip in red algae was at 672 nm in January and at 677 nm in May. The seasonal spectral reflectance shift may be due to varying ratio of Chl a in PS I and PS II between seasons. The brown algae have a similar reflectance dip in January and May at 664 nm and 663 nm respectively, partly due to absorbance impact of Chl c (peak at 630 nm).

Pigment composition and concentration is also important for interpretation of *in-situ* monitoring using UHI as they determine the spectral reflectance signature of macroalgae [outlined in 26]. For example, Chl a has been widely used to determined phytoplankton biomass^56^. With UHI mapping, pigment composition, allows us to differentiate between pigment groups (green, red, and brown algae)^26^. This study provides additional information regarding the health state in terms of photophysiological and physical state of the species present. In conclusion, we show that the examined macroalgae were growing new tissue during the Polar Night that they are photophysiologically functioning. High E_K_ and low F_v_/F_m_ in kelp species indicate a PS II with slower activity during the Polar Night relative to the end of the light season. We also demonstrate how the *in-vivo* Chl a emission spectra, which reflect the light energy transferred to PS II in all species, could be interpreted. The *in-vivo* absorbance spectra were used to interpret the *in-vivo* Chl a emission spectra to determine the ratio of [Chl a] in PS I vs PS II. We acknowledge that more research and experimentation is needed to further investigate reported seasonal variation in emission intensity. We also measured a spectral shift in the *in-vivo* Chl a absorbance and reflectance peaks in the red part of the visible spectrum, giving us additional confirmation about whether the Chl a are bonded to PS I or PS II. This study provides evidence of tissue growth and indication of photophysiological activity in red, green, and brown macroalgae during the Polar Night, complementing biodiversity and areal cover information gathered through ROV-UHI mapping of kelp forest habitat at the same site and time as this study^26^. Combining photophysiology and health state with UHI habitat mapping also provides an overview of the study site at different time and spatial scales which may be important in future identification, mapping and monitoring of underwater habitats.

## Data Availability

Light data are available through open access through NIRD (National Infrastructure for Research Data). The data are published in datasets covering annual time series (2020) from spectroradiometer (raw data and E_PAR_ data): Berge, J., et al. USSIMO Spectroradiometer Raw Data Time Series (2020) Measured under the Dome of a Light Observatory in the Arctic (Ny-Ålesund, Svalbard, Norway) [Data Set], (Norstore, 2021). Data from the all-sky camera can be found: Johnsen, G., et al. Pictures from an All-Sky Camera with Hourly Resolution from the Light Observatory at Ny-Ålesund, Svalbard, Norway (Complete Year 2020) [Data Set], (Norstore, 2021). Other datasets generated and analyzed during the current study are available from the corresponding author on reasonable request.
